# Multidisciplinary Progress in Obesity Research

**DOI:** 10.3390/genes13101772

**Published:** 2022-09-30

**Authors:** Xiaoqing Lu, Yuxin Jin, Dexin Li, Jingxin Zhang, Jingyan Han, Yin Li

**Affiliations:** 1 Department of Integration of Chinese and Western Medicine, School of Basic Medical Sciences, Peking University, Beijing 100191, China; 2 Tasly Microcirculation Research Center, Peking University Health Science Center, Beijing 100191, China; 3 Key Laboratory of Stasis and Phlegm, State Administration of Traditional Chinese Medicine of the People’s Republic of China, Beijing 100191, China; 4 Beijing Laboratory of Integrative Microangiopathy, Beijing 100191, China; 5 State Key Laboratory of Core Technology in Innovative Chinese Medicine, Beijing 100191, China

**Keywords:** obesity, cancer, nervous system diseases, multidisciplinary treatment of obesity, metabolic diseases

## Abstract

Obesity is a chronic disease that endangers human health. In recent years, the phenomenon of obesity has become more and more common, and it has become a global epidemic. Obesity is closely associated with many adverse metabolic changes and diseases, such as insulin resistance, type 2 diabetes mellitus, coronary heart disease, nervous system diseases and some malignant tumors, which have caused a huge burden on the country’s medical finance. In most countries of the world, the incidence of cancer caused by obesity is increasing year on year. Diabetes associated with obesity can lead to secondary neuropathy. How to treat obesity and its secondary diseases has become an urgent problem for patients, doctors and society. This article will summarize the multidisciplinary research on obesity and its complications.

## 1. Epidemiology and the Financial Burden of Obesity

Obesity represents a condition of chronic excess fat mass. Several epidemiological studies have revealed an alarming increase in the number of obese individuals worldwide [1]. Since 1980, the rate of overweight and obesity has doubled, and now nearly one-third of the world’s population is overweight or obese [2,3]. In 2016, more than 1.9 billion individuals aged at least 18 years were overweight (with a BMI of ≥25 kg/m^2^), of which more than 650 million had a BMI ≥ 30 kg/m^2^. Worldwide rates of obesity increased almost three-fold between 1975 and 2016 [4]. In the past 40 years, overweight and obesity have increased rapidly. According to Chinese standards, the latest national prevalence estimates for 2015–19 are 6.8% and 3.6% for children under 6 years of age, 1% and 7.9% for children and adolescents aged 11 to 17 years of age, respectively. Adults (≥18 years of age) were 34.3% overweight and 16.4% obese. Obesity has become a major public health problem in China [5]. Obesity is associated with many diseases, and is closely related to diseases, such as diabetes [6], cardiovascular disease [7], cancer [8], musculoskeletal disorders and psychological disorders [7]. Four million deaths worldwide in 2015 were associated with excess weight, and in 2014, the number was more than 320,000 in the United States [9]. Morbidity due to obesity-related cancer is increasing year on year in most countries around the world and represents a threat to human health [10,11].

The treatment of obesity and its related diseases can carry a heavy financial burden [12]. In addition to direct healthcare costs, there are other losses such as missed work and reduced household income. The economic burden due to obesity in the United States was approximately USD 1.4 trillion in 2017 [13]. Relevant data show that diabetic peripheral neuropathy (DPN) in the United States costs tens of billions of dollars each year and the annual medical costs are rising year on year [14]. The Swedish Obesity Study showed that obese people were three times more likely to receive disability pensions, have twice as many sick days and have higher annual medication costs than non-obese people [15]. Research by the McKinsey Global Institute (MGI) showed that the total economic impact of obesity is approximately USD 2 trillion per year, or 2.8% of the world GDP, roughly equivalent to the economic cost of smoking or armed violence, war and terrorism [16].

## 2. Characteristic of Obesity

The World Health Organization (WHO) has defined obesity as a BMI ≥ 30 kg/m^2^, an epidemic and a disease within itself, which is chronic and progressive in nature with the potential for relapse [4]. Overweight and obesity are characterized by excess body fat, and those with a body mass index (BMI) ≥ 25 or ≥30 kg/m^2^ are considered obese [17]. Obesity is a multifactorial disease caused by a chronic positive energy balance, when energy intake exceeds energy expenditure, excess energy is converted to triglycerides, which are stored in adipose tissue depots and the volume of adipose tissue expands, thereby increasing body fat and leading to weight gain. The current passive overconsumption of energy-dense, nutrient-poor foods and beverages has been identified as a major driver of the obesity epidemic [7,18]. Decreased physical activity due to modern lifestyles is also an important contributor [19]. A higher BMI is typically associated with higher all-cause mortality and cardiovascular disease mortality, with a gradual increase as BMI increases above 25 kg/m^2^ [20].

Obesity is characterized by a state of low-grade systemic inflammation or “metabolic inflammation.” Adipocytes produce and secrete several proteins called adipokines, which play an important role in inflammation. These adipokines include TNF-α, leptin, resistin, visfatin, IL-6, and adiponectin. There are more than 50 known adipokines, which mainly differ in their role in inflammation. The adipose tissue of obese people mainly secretes proinflammatory adipokines, while lean people secrete anti-inflammatory adipokines [21]. Chronic inflammation has been shown to underlie the promotion of tumor growth [22]. The presence of inflammatory cells and their mediators in tumor tissue induces cell proliferation and migration, and contributes to angiogenesis [23].

## 3. Obesity as a Risk Factor for Other Diseases

Obesity has been listed by the WHO as one of the top ten risk factors that threaten human health. Obesity has adverse effects on many physiological functions and poses a significant public health threat. In addition to increased mortality, obesity and increased central fat distribution are associated with increased diseases. More than 230 obesity-related comorbidities and complications have been identified [24]. It is important to emphasize that obesity causes changes in the body’s physiological and hormonal environments and represents a risk factor for the onset of different metabolic disorders, such as type 2 diabetes, as well as the development of cardiovascular diseases [25]. Moreover, it has been well established that the risk of many types of malignancies is increased in obese individuals [26].

Type 2 diabetes is strongly associated with obesity, with more than 80% of type 2 diabetes attributable to obesity, which also contributes to many diabetes-related deaths [27]. Weight gain after the age of 18 years for women and 20 years for men also increases the risk of type 2 diabetes [28]. The adverse effects of obesity on blood lipids include high concentrations of serum cholesterol, low-density lipoprotein (LDL) cholesterol, very-low-density lipoprotein (VLDL) cholesterol and triglycerides, as well as an approximately 5% decrease in serum high-density lipoprotein (HDL) cholesterol concentrations [29]. Central fat distribution also has an important effect on dyslipidemia. Abundant evidence shows that patients with obesity and lipodystrophy have similar clinical health conditions, namely hypertriglyceridemia, insulin resistance, and fatty liver [30,31]. It is estimated that the number of obese and overweight people worldwide exceeds the number of undernourished people [32]. Obesity is a chronic disease [33]. Its etiology lies not only in the abnormal increase in body fat, but also in the release of excessive adipokines from adipose tissue. These adipokines in the blood can bind to specific receptors on the surface of target cells, thereby affecting the metabolism of tissues and organs. For example, adipokines and cytokines may reduce the insulin sensitivity of tissues and lead to the occurrence and development of diabetes [32].

Obese people usually have high blood pressure. The risk of hypertension is highest in those with upper body and abdominal obesity. Obesity is strongly associated with an increased risk of coronary heart disease, heart failure, myocardial steatosis, and atrial fibrillation [34]. For every 1 unit increase in BMI in men and women, the risk of atrial fibrillation increases by 4% [35]. Not only is obesity strongly associated with more general coexisting risk factors for coronary heart disease (such as coronary artery disease, hypertension, diabetes, and obstructive sleep apnea), but obesity itself also affects myocardial structure and pump performance [36].

Being overweight can increase the risk of many cancers, and in 2014, approximately 40% of cancers in the United States were associated with overweight and obesity [37]. Overweight and obesity can also increase the risk of death from cancer. The mechanisms underlying the increased risk of cancer development and death include changes in sex hormone metabolism, altered levels of insulin and insulin-like growth factors and altered adipokine pathways [38,39]. Obesity increases the risk of gallbladder disease and non-alcoholic fatty liver disease, and obesity affects the hepatobiliary system mainly by causing cholelithiasis [40]. Additionally, obesity is a risk factor for gastrointestinal diseases such as gastroesophageal reflux disease, erosive esophagitis, esophageal adenocarcinoma and gastric cancer [8].

Obese women have a higher lifetime rate of failure to deliver and failure to conceive, often associated with irregular menstruation and anovulatory cycles. Obese pregnant women are more likely to develop multiple maternal and perinatal complications, and these risks increase with maternal obesity [41]. Obesity has a similar effect on men. In men, obesity adversely affects spermatogenesis and testosterone production by suppressing the hypothalamic pituitary axis [42]. In addition, the testosterone-to-estradiol ratio is reduced in infertile obese men due to increased aromatase activity caused by obesity, and this ratio is improved when aromatase inhibitors are used, promoting spermatogenesis [43]. Ovulation dysfunction is more common in obese women. Much of this ovulatory dysfunction can be confused with a diagnosis of polycystic ovary syndrome (PCOS). The risk of anovulatory infertility increases as BMI increases [44].

Obese people often suffer social stigma because of their weight. This stigma is reflected in education, employment and healthcare. Obese people also suffer from discrimination in employment, especially in physically demanding occupations [45]. Depression is associated with severe obesity, especially in younger patients and in women [46].

## 4. Obesity and Cancer

Recent evidence indicates that obesity is an established risk factor for malignancies [47,48] in addition to the well-established cancer risk factors such as genetic predisposition, ionizing radiation, tobacco use, infections, unhealthy diet, alcohol consumption, sedentary lifestyle, and other environmental exposures, and excess adiposity is associated with approximately 20% of all cancers [49]. According to the International Agency for Research on Cancer (IARC) Working Group, there is convincing evidence that excess body weight is associated with an increased risk of cancer in at least 13 anatomic sites, including endometrial, esophageal, renal and pancreatic adenocarcinomas, hepatocellular carcinoma, gastric cardia cancer, meningioma, multiple myeloma, colorectal, postmenopausal breast, ovarian, gallbladder and thyroid cancers [8]. Based on a different classification of the strength of evidence for the link between overweight/obesity and cancer risk, the World Cancer Research Fund/American Institute for Cancer Research (WCRF/AICR) found in common with the IARC Working Group, convincing and sufficient evidence for cancers of the endometrium, esophagus (adenocarcinoma), colon and rectum, liver, pancreas, postmenopausal breast and kidney (renal adenocarcinoma) [50].

Although a large evidence base on excess body weight and body size and cancer risk has been accumulated, some nuances of these associations still warrant further investigation. The key remaining etiologic question is whether weight distribution or total body size is more important in cancer risk. Mendelian randomization to analyze the relationship between adiposity and cancer was studied, and this showed a strong causal relationship between obesity and colorectal cancer [51]. A recent systematic review and meta-analysis examining anthropometric factors and endometrial cancer risk noted a limited number of studies reported on waist circumference (WC) (4 out of 30) and waist-to-hip ratio (WHR) (5 out of 30). Variables such as SNPs associated with BMI, WHR and birth weight were used. Although few studies performed mutual adjustments for BMI and WC to elucidate their independent role in endometrial cancer risk [52], it is plausible that measures of body fat distribution, such as WC and WHR, could be stronger determinants of cancer risk than overall body size, as WC and WHR have been shown to be better predictors of morbidity and all-cause mortality than BMI [53].

Notably, body fatness in childhood and adolescence is inversely related to the risk of premenopausal breast cancer as well as postmenopausal breast cancer, suggesting a long-term effect of body fatness at a young age on breast cancer risk in later life. These findings contrast with the higher risk of breast cancer among postmenopausal women [54] who have greater body fatness in adulthood. Early life, including childhood and adolescence, is hypothesized to be a critical window for breast carcinogenesis. This is a period of rapid growth and development of breast tissue, with higher rates of mammary gland tissue proliferation during puberty, which may increase susceptibility to molecular damage and may explain why particular exposures may be important for breast cancer risk later in life. Sex hormones may also partly explain the inverse relationship between adiposity in early life and the risk of breast cancer. Overall, the mechanisms underlying the inverse association between early life body fatness and breast cancer risk are complex, and are likely multiple and not well-delineated.

In recent years, genetics have also been shown to play a role in obesity. Fat mass and obesity-associated (FTO) single-nucleotide polymorphism sites (SNPs) have been firmly associated with increased BMI and higher risks of various types of cancers in people of multiple races, and play a role in the appetite control and the overexpression of this gene is linked to increased food intake. The role of FTO SNPs in the development of obesity and cancer has been gradually revealed [55,56,57,58]. It is reported that mutations in the *FTO* gene raise blood levels of leptin, a known mediator or growth factor between obesity and colon cancer, which activates a variety of pathways associated with colon cancer [59]. In addition, leptin has been suggested as an intermediate link between obesity and breast cancer [60]. Intriguingly, the mammalian target of rapamycin is also one of the signal mediators of obesity-related factors, such as leptin, adiponectin, and inflammatory cytokines, through the AMPK pathways [61].

In particular, growing interest has recently been placed on the role of adipose tissue-secreted molecules in the development of cancer [62]. White adipose tissue, once regarded as the major site of energy storage and homeostasis, is now known to be an endocrine organ producing numerous biologically active molecules and hormones [63], and secreting different types of molecules called adipokines, which are implicated in the pathogenesis of numerous types of malignancies [62,64]. One of the most important is adiponectin, which is mainly secreted by adipocytes, and is also produced, to some extent, by bone marrow, osteoblasts, fetal tissue, myocytes, cardiomyocytes and salivary gland epithelial cells [65,66].

In obese individuals, the levels of pro-inflammatory adipokines are elevated, whilst the levels of anti-inflammatory adipokines are reduced. In addition, there are altered levels of chemokines and cytokines [67]. Adiponectin orchestrates multiple biological functions to inhibit cancer progression, and has been shown to inhibit cell proliferation via the ERK1/2-MAPK pathway in T47D cells and inhibited secondary tumor development in adjacent fat pads [68]. Adiponectin administration to a parent cell line suppressed cell migration and tube formation and induced cell cycle arrest, while adiponectin deficiency enhanced the proliferative, migratory and pro-angiogenic potential of these cells [69]. It was demonstrated that adiponectin overexpression in prostate cancer cells results in the depletion of vascular endothelial growth factor A (VEGFA) and vice versa via an AMPK/TSC2-mediated mechanism [70]. In addition, adiponectin induced cell cycle arrest and apoptosis in cancer cells via the AMPK/FoxO3A axis [71] and apoptosis in hepatocellular carcinoma through the differential modulation of thioredoxin proteins [72]. In models of colorectal cancer, adiponectin knockdown resulted in the increased multiplicity of colorectal polyps which were also more aggressive and metastatic with higher cyclooxygenase 2 levels compared to their wild-type counterparts, suggesting that higher levels of circulating adiponectin could also be associated with the better prognosis of colorectal cancer. In adiponectin-deficient mice, adiponectin inhibited tumor progression and angiogenesis when fed an obesogenic diet but not with a normal diet [73,74]. It was demonstrated that adiponectin conferred protection against inflammation-induced colon cancers by preventing the apoptosis of goblet cells and promoting the differentiation of epithelial cells to goblet cells [75,76]. Moreover, adiponectin represses colon cancer cell proliferation via AdipoR1- and -R2-mediated AMPK activation [77] and it has been reported to result in a 27% lower risk of developing colorectal cancer [78]. Due to its strong negative association with multiple cancers and its role in tumor angiogenesis and vasculature development, many research groups have studied the involvement of adiponectin in cancer invasion, migration and metastasis. Adiponectin has been shown to counteract the effect of leptin by inhibiting leptin-induced migration and invasion in breast cancer [79]. Mechanistically, adiponectin prevents leptin-induced invasion by inhibiting signal transducer and activator of transcription 3 (STAT3) phosphorylation and MAPK-mediated nuclear translocation [80]. Adiponectin inhibits hepatic stellate cell activation, intratumoral macrophage infiltration and diminishes tumor vascularization by downregulating ROCK/IP10/VEGF signaling and the inhibition of lamellipodia formation [81]. Adiponectin upregulates epithelial marker expression and decreases mesenchymal markers, which can be reversed by knocking down Twist, AdipoR1 and AdipoR2 [82].

In addition, obesity may very well cause insulin resistance through promoting chronic inflammation in adipose tissue, and by increasing insulin secretion in the system, thereby activating multiple growth pathways [83]. However, insulin per se can also cause obesity due to its nature as a potently anabolic hormone. Chronic inflammation, excess insulin secretion and the hyperactivation of growth pathways are closely associated with tumor development and progression [84]. Obesity can influence the tumor microenvironment through dysfunctional adipose tissue and altered extracellular signals. During obesity, vascular dysfunction is impaired and creates pockets of hypoxia. Adipose tissue hypoxia establishes a highly proinflammatory microenvironment, which is a favorable microenvironment for tumor promotion [85]. Dysfunctional adipose tissue leads to dysregulated adipokine production, preferentially releasing proinflammatory adipokines [86]. This adipokine imbalance is closely associated with insulin resistance, lipolysis and pro-inflammatory signaling pathways, contributing to a favorable microenvironment for tumor growth and progression. In addition, macrophage infiltration into obese adipose tissues promotes ECM remodeling through the elevation of several ECM components, thereby providing a tumor-friendly environment [87].

## 5. Obesity-Associated Diabetes and Neurological Diseases

Diabetes is a chronic disease, which is caused by the inability of the body to produce enough insulin or the inability to use insulin properly. At the same time, diabetes is one of the main causes of many serious health problems, including neurological diseases [88]. Clinical observations, epidemiological evidence and animal disease models strongly indicate that metabolic syndrome (including obesity, dyslipidemia and type 1 and type 2 diabetes) is associated with an increased risk of peripheral neuropathy. Metabolic syndrome neuropathy preferentially affects small unmyelinated axons, but may also affect autonomic nerves and large fibers [89]. Neuropathic pain and hypoesthesia can lead to a range of undesirable consequences, including falls, impaired quality of life, restricted activities of daily living and depressive symptoms [90].

The most common type of diabetic peripheral nervous system disorder is called bilateral and symmetric hand–foot nerve injury. Patients will experience sensory disturbances in the early stages of illness, including the loss of sensory or spontaneous touch, vibration, tingling, hot and cold pain and other sensations. As the course of the disease continues, patients will develop motor weakness and multiple organ dysfunction, which are caused by motor and autonomic nerve damage [91,92]. Among the various forms of diabetic neuropathy, distal symmetrical polyneuropathy and diabetic autonomic neuropathy, especially cardiovascular autonomic neuropathy (CAN), are currently the most studied [14]. In more than half of diabetic patients, peripheral and autonomic nervous system damage is a common phenomenon in clinical diabetic complications. The specific cause is due to diffuse and focal nervous system damage, called diabetic neuropathy [93]. The proposed theories on diabetic neuropathy include metabolism, neurovascular and autoimmune pathways. In addition, mechanical compression (such as carpal tunnel), genetic, social and lifestyle factors (such as long-term drinking and smoking) are all related to diabetic neuropathy [94]. The pathogenesis and diagnostic criteria of diabetic neuropathy are not yet clear. Systemic and cellular disorders in glucose and lipid metabolism are believed to be involved in the pathogenesis, including increased oxidative stress, activation of polyol and protein kinase C pathways and the involvement in the activation of damaged genes, etc. [95].

In diabetic patients, high blood sugar can cause damage to small blood vessels, which in turn impairs the supply of oxygen and nutrients to nerve cells. At the level of disease development, the first stage is damage to the distal sensory and autonomic nerve fibers, and then the damage gradually leads to the loss of the protective sensation of the skin and foot joints as the disease progresses, and half of the DPN may be asymmetric [94,96,97]. Diabetic neuropathy is related to multiple risk factors such as hemoglobin A1c (HbAlc) levels, high blood pressure, smoking status and BMI [98]. A study recruited 1441 participants with type 1 diabetes, and after an average follow-up of 23 years, 33% of the participants developed DPN and 44% of the participants developed CAN. At the start of the follow-up, 90% of the participants had neither DPN nor CAN; 5% had DPN only; 4% had CAN only; and 1% had both [99]. No effective treatment for diabetic neuropathy has been developed to date, and the main clinical strategy is still the control of blood sugar. Intensified blood sugar control can reduce the risk of DPN and CAN [99]. Depending on age, the diabetes course, blood sugar control and the difference between type 1 and type 2 diabetes, the prevalence of peripheral neuropathy in diabetic adults is estimated to be 6–51%, and the clinical manifestations are varied, from asymptomatic to painful neuropathy [90]. Some researchers have evaluated the influencing factors of peripheral neuropathy in patients with type 2 diabetes using an evidence-based medicine system. The results show that the course of diabetes, age, HbA1c and diabetic retinopathy (DR) are associated with a significant increase in the risk of peripheral neuropathy in diabetic patients, while BMI and smoking, total triglycerides and total cholesterol did not show any increase in the risk of peripheral neuropathy in diabetic patients [100].

With regard to whether combined exercise training improves DPN, the experimental results show that after eight weeks of combined (resistance-aerobic) exercise training, serum kinesin-1 levels and aerobic endurance decrease, but this decline is not statistically significant. The upper body strength increased but was not significant, while the lower body showed a significant increase in strength. Regarding the gradual nature of DPN, it seems that even small changes produced by combined exercise training are useful. Nevertheless, more research is needed in this area [101]. Dietary interventions can effectively improve diabetic neuropathy in animal models, and promising data indicate that they may also improve diabetic neuropathy in humans [102]. Recent studies have shown that mitochondrial dysfunction is the basis for the occurrence and development of neuropathy in prediabetes and diabetic patients. Hyperglycemia and dyslipidemia contribute to the pathogenesis of neuropathy, but have different effects on neuronal mitochondrial dynamics and function. Mitochondrial bioenergetics, which are damaged in pre-diabetes or diabetes, plays a key role in the progression of neuropathy by reducing axon energy and causing the inability to maintain neuronal function. To compensate for this reduction in bioenergy, dorsal root ganglion (DRG) neurons undergo mitochondrial biogenesis in the event of hyperglycemia or proton leakage during dyslipidemia. Elevated saturated fatty acids (LCSFAs) associated with dyslipidemia can impair mitochondrial transport, depolarize the 1-Methyl-4-phenylpyridinium (MPP), induce ATP loss and provoke neuronal apoptosis [103].

Diabetic neuropathy is also related to genetics. V. Spallone et al. summarized the results of animal and human studies linking microRNAs (miRNAs) to diabetic neuropathy, and explored the possible pathogenic significance of these associations, especially with regard to miR-128a, miR-155a and miR-499a, and their application in the screening of diabetic neuropathy. The authors found that the miRNA gene polymorphism is closely related to diabetic neuropathy [104]. Axon degeneration is the main pathological feature of many early peripheral neuropathies, including diabetic neuropathy. Yalan Cheng et al. studied whether Sarm1−/− mice were resistant to streptozotocin (STZ)-induced diabetes, diabetic neuropathy and the underlying molecular mechanism. It was found that Sarm1−/− mice had no resistance to STZ-induced diabetes, but had resistance to STZ-induced diabetic neuropathy. In addition, the Sarm1 gene defect significantly reduced the changes in the gene expression profile induced by STZ in the sciatic nerve [105]. There is evidence that an association exists between low vitamin D levels and diabetic neuropathy. P. Shillo and others have proved that the vitamin D level in people with painful DPN is significantly different to that in healthy people, indicating that vitamin D is involved in the pathogenesis of DPN. Vitamin D deficiency is also associated with high blood pressure, abnormal blood sugar and abnormal blood lipids, which are considered to be independent risk factors for urinary peripheral neuropathy [106].

Type 1 diabetes mellitus (T1DM) neuropathy and T2DM neuropathy are fundamentally different. In T1DM, glucose control has a great impact on the prevention of neuropathy. Therefore, future efforts should continue to focus on this treatment approach. In contrast, in T2DM, glucose control has little effect on the prevention of neuropathy, and more research is needed to determine the underlying mechanism of neuropathy development [107].

## 6. Multidisciplinary Treatment of Obesity

The most important therapies are diet and lifestyle intervention, which require support and help based on sports, medicine and nutrition. The treatment of obesity is based on improving the structure of the daily diet, strictly controlling the intake of calories and strengthening regular exercise [108]. A low-fat, low-carbohydrate and high-protein diet or the Mediterranean diet is an important means of achieving weight loss, which can prevent malnutrition, muscle loss and osteoporosis while controlling an extremely low calorie intake [109]. This type of diet is beneficial for human metabolism, but may not be suitable for children, adolescents or the elderly [110]. In addition, replacing one or two meals with dietary supplements may help maintain a balanced diet and improve the compliance rate of dietary schedules [111]. The diet also needs to match the patient’s personal food preferences, lifestyle and medical conditions [112]. As important as the improvement of dietary structure, physical exercise is the lifestyle intervention method recommended by almost all scientific guidelines, which includes aerobic exercise for at least 150 minutes a week and muscle resistance training at least 3 times a week. An increase in physical activity can reduce abdominal fat, increase muscle glucose tolerance and insulin sensitivity. In addition to being conducive to physical health, moderate exercise is also conducive to mental health and may reduce depression [113,114]. Rapid and large-scale muscle and fat free weight loss may also lead to malnutrition and osteoporosis. Therefore, attention should be paid to body composition such as muscle-fat distribution and bone health. Both should be examined at least 2 years after surgery [115].

Drug therapy is the most direct and effective way to lose weight. Several common pathways and pharmacological targets can centrally or peripherally regulate lipid metabolism, such as the leptin–melanocortin axis, the opioid system and the glucagon-like peptide-1 (GLP-1)/GLP-1 system [116,117]. A recent meta-analysis of new anti-obesity drugs and their effects on weight loss showed that the following drugs reduced body weight percentage compared to placebo for at least 12 months, with an overall range of 2.9–6.8% [118]. Compared with other current drugs, Phentermine/Topiramate has a higher probability of reaching the predefined threshold of clinically meaningful weight loss. There was no difference in the rates of treatment discontinuation due to drug-related adverse events in patients treated with Phentermine/Topiramate, Liraglutide, and Naltrexone-Bupropion. However, compliance with Lorcaserin and Orlistat is low, and there is a relatively high proportion of adverse drug reactions [119]. Clinically, there are also differences in the drugs selected in specific patient groups. For example, Liraglutide has hypoglycemic effects and may be more suitable for patients with diabetes [120]. The combination of Naltrexone-Bupropion may cause chronic drug dependence [121]. Historically, concerns about the long-term safety of drug therapy for weight loss have limited the clinical use of drugs with significant adrenergic effects or drugs with central appetite-suppressive effects [122]. The combination of drugs in various complementary pathways may potentially promote weight loss in various patients. The development of advanced subcutaneous delivery technology provides an opportunity to develop breakthrough peptides and other biological agents for the treatment of obesity. Drug combinations that target multiple, complementary sites have the potential to promote double-digit weight loss in a broader, heterogeneous patient population. Furthermore, the development of advanced subcutaneous delivery technologies has opened up opportunities for the development of breakthrough peptide and biologic agents for the treatment of obesity [116].

Bariatric surgery is an established and effective part of weight loss management for morbidly obese patients. It is applicable in obese patients with a BMI ≥ 40 kg/m^2^ or in individuals with a BMI > 35 kg/m^2^ in the presence of type 2 diabetes or other major complications [123]. When non-surgical weight loss methods have been exhausted, weight loss surgery has become the choice of severely obese patients. In addition to the direct impact on weight loss, bariatric surgery also improved many health indicators in the postoperative period. For example, physical functioning, metabolic parameters and autonomic nervous system modulation, more specifically, energy expenditure, physical activity level, muscular strength and peak oxygen consumption are improved [124].

In addition, complementary and alternative therapies (CATs) also have encouraging effects in the treatment of obesity. The National Center for Complementary and Alternative Medicine (NCCAM) of the United States divides CATs into five categories, which are based on biology, energy, manipulation and body, psychosomatic therapy and the whole medical system. In weight loss, the most important are oral vitamins, herbal extracts, food and dietary supplements, followed by acupuncture and hypnotherapy [125]. There are many types of dietary supplements on the market, but the quality of these supplements is mixed. A study found that the risk of adverse events increased after chitosan and fiber supplementation. There have been many experiments and reviews conducted on Chinese herbal medicine, especially focusing on herbs that are most commonly used in weight loss preparations [126]. A significant decrease in weight, body fat and waist or hip circumference was observed following treatment with xin-ju-xiao-gao-fang (XJXGF), yellow pea fiber, bofu-tsusho-san, RCM-104, pistachio, Satiereal^®^, Monoselect Camellia, Linggui Zhugan Decoction, Pu’er tea, pistachio, or Monoselect Camellia, Satiereal and Catechin enriched green tea [127]. Manual acupuncture is a traditional Chinese medical practice which involves inserting small needles into acupoints on the skin. Research has found that two groups of acupoints have the best effect on weight loss, which are ST36-CV12-ST25-SP6 and ST40-ST24-SP15-ST37-CV4 [128]. There are several possible mechanisms for this effect, including increased metabolism, regulation of obesity-related neuropeptides and reduced triglyceride and cholesterol levels [129]. In addition, ear acupuncture, or auricular needling is also a weight loss therapy commonly used in modern Chinese medicine. The commonly used acupoints are “Endocrine”, “Stomach”, “Spleen”, “Shenmen”, “Hunger point”, “Sanjiao”, “Lung” and “Subcortex”. The employed auricular acupoint therapy mainly includes auricular acupressure, ear acupuncture, auricular vagus nerve stimulation and auricular laser radiation [130].

## 7. Conclusions

As a chronic disease, obesity has gradually become a major public health problem faced by many countries in the world, and it is one of the most important health challenges in the future. Moreover, obesity is not only a simple disease of overweight or excess body fat. How to treat obesity and its complications and secondary diseases has become an urgent problem for patients, doctors and society. At present, the treatment of obesity includes lifestyle intervention, drug treatment, bariatric surgery, complementary medicine and alternative therapy. The combination of multiple treatment methods and multidisciplinary solutions are conducive to improving the efficiency of obesity treatment, with good curative effect and great demand among patients. Multidisciplinary treatment also has higher economic value, which is conducive to reducing the increasingly severe public health challenges caused by obesity. With regard to the differences in safety, efficacy and response to treatment, the ideal weight loss method should be highly individualized, and appropriate candidates for drug treatment, behavioral intervention and surgical intervention should be determined.

## Data Availability

Not applicable.

## References

[1] Di Cesare M., Bentham J., Stevens G.A., Zhou B., Danaei G., Lu Y., Bixby H., Cowan M.J., Riley L.M., Hajifathalian K. (2016). Trends in adult body-mass index in 200 countries from 1975 to 2014: A pooled analysis of 1698 population-based measurement studies with 19.2 million participants. Lancet.

[2] Ataey A., Jafarvand E., Adham D., Moradi-Asl E. (2020). The relationship between obesity, overweight, and the human development index in world health organization eastern mediterranean region countries. J. Prev. Med. Public Health.

[3] Lin X., Li H. (2021). Obesity: Epidemiology, pathophysiology, and therapeutics. Front. Endocrinol..

[4] WHO (2020). World Health Organization (WHO): Obesity and Overweight.

[5] Pan X.F., Wang L., Pan A. (2021). Epidemiology and determinants of obesity in china. Lancet Diabetes Endocrinol..

[6] Singh G.M., Danaei G., Farzadfar F., Stevens G.A., Woodward M., Wormser D., Kaptoge S., Whitlock G., Qiao Q., Lewington S. (2013). The age-specific quantitative effects of metabolic risk factors on cardiovascular diseases and diabetes: A pooled analysis. PLoS ONE.

[7] Swinburn B.A., Sacks G., Hall K.D., McPherson K., Finegood D.T., Moodie M.L., Gortmaker S.L. (2011). The global obesity pandemic: Shaped by global drivers and local environments. Lancet.

[8] Lauby-Secretan B., Scoccianti C., Loomis D., Grosse Y., Bianchini F., Straif K. (2016). Body fatness and cancer—Viewpoint of the iarc working group. N. Engl. J. Med..

[9] Afshin A., Forouzanfar M.H., Reitsma M.B., Sur P., Estep K., Lee A., Marczak L., Mokdad A.H., Moradi-Lakeh M., Naghavi M. (2017). Health effects of overweight and obesity in 195 countries over 25 years. N. Engl. J. Med..

[10] Ng M., Fleming T., Robinson M., Thomson B., Graetz N., Margono C., Mullany E.C., Biryukov S., Abbafati C., Abera S.F. (2014). Global, regional, and national prevalence of overweight and obesity in children and adults during 1980–2013: A systematic analysis for the global burden of disease study 2013. Lancet.

[11] Siegel R.L., Jemal A., Wender R.C., Gansler T., Ma J., Brawley O.W. (2018). An assessment of progress in cancer control. CA A Cancer J. Clin..

[12] Wang Y.C., McPherson K., Marsh T., Gortmaker S.L., Brown M. (2011). Health and economic burden of the projected obesity trends in the USA and the uk. Lancet.

[13] Waters H., DeVol R. (2021). Weighing down America: The Health and Economic Impact of Obesity.

[14] Pop-Busui R., Boulton A.J., Feldman E.L., Bril V., Freeman R., Malik R.A., Sosenko J.M., Ziegler D. (2017). Diabetic neuropathy: A position statement by the american diabetes association. Diabetes Care.

[15] Narbro K., Agren G., Jonsson E., Näslund I., Sjöström L., Peltonen M. (2002). Pharmaceutical costs in obese individuals: Comparison with a randomly selected population sample and long-term changes after conventional and surgical treatment: The sos intervention study. Arch. Intern. Med..

[16] Dobbs R., Sawers C., Thompson F., Manyika J., Woetzel J.R., Child P., McKenna S., Spatharou A. (2014). Overcoming Obesity: An Initial Economic Analysis.

[17] World Health Organization Obesity and Overweight. Fact Sheet No. 311. January 2015; Volume 13. http://www.who.int/mediacentre/factsheets/fs311/en/Stand.

[18] Yuan F., Zhang Q., Dong H., Xiang X., Zhang W., Zhang Y., Li Y. (2021). Effects of des-acyl ghrelin on insulin sensitivity and macrophage polarization in adipose tissue. J. Transl. Int. Med..

[19] Ladabaum U., Mannalithara A., Myer P.A., Singh G. (2014). Obesity, abdominal obesity, physical activity, and caloric intake in us adults: 1988 to 2010. Am. J. Med..

[20] McTigue K., Larson J.C., Valoski A., Burke G., Kotchen J., Lewis C.E., Stefanick M.L., Van Horn L., Kuller L. (2006). Mortality and cardiac and vascular outcomes in extremely obese women. JAMA.

[21] Khanna D., Khanna S., Khanna P., Kahar P., Patel B.M. (2022). Obesity: A chronic low-grade inflammation and its markers. Cureus.

[22] Deng T., Lyon C.J., Bergin S., Caligiuri M.A., Hsueh W.A. (2016). Obesity, inflammation, and cancer. Annu. Rev. Pathol..

[23] Hanahan D., Coussens L.M. (2012). Accessories to the crime: Functions of cells recruited to the tumor microenvironment. Cancer Cell.

[24] Rueda-Clausen C.F., Ogunleye A.A., Sharma A.M. (2015). Health benefits of long-term weight-loss maintenance. Annu. Rev. Nutr..

[25] Lu Y., Hajifathalian K., Ezzati M., Woodward M., Rimm E.B., Danaei G., Selmer R., Strand B.H., Dobson A., Hozawa A. (2014). Metabolic mediators of the effects of body-mass index, overweight, and obesity on coronary heart disease and stroke: A pooled analysis of 97 prospective cohorts with 1·8 million participants. Lancet.

[26] Renehan A.G., Tyson M., Egger M., Heller R.F., Zwahlen M. (2008). Body-mass index and incidence of cancer: A systematic review and meta-analysis of prospective observational studies. Lancet.

[27] Aras M., Tchang B.G., Pape J. (2021). Obesity and diabetes. Nurs. Clin. N. Am..

[28] Kleinert M., Clemmensen C., Hofmann S.M., Moore M.C., Renner S., Woods S.C., Huypens P., Beckers J., De Angelis M.H., Schürmann A. (2018). Animal models of obesity and diabetes mellitus. Nat. Rev. Endocrinol..

[29] Poirier P., Giles T.D., Bray G.A., Hong Y., Stern J.S., Pi-Sunyer F.X., Eckel R.H. (2006). Obesity and cardiovascular disease: Pathophysiology, evaluation, and effect of weight loss. Arterioscler. Thromb. Vasc. Biol..

[30] Pivtorak K.V., Shevchuk N.A., Pivtorak N.A., Fedzhaga I.V. (2019). Correction of adipocyte secretion disorders in patients with non-alcoholic fatty liver disease with overweight and obesity. Wiad. Lek. (Wars. Pol. 1960).

[31] Serradilla Martín M., Oliver Guillén J.R., Palomares Cano A., Ramia Ángel J.M. (2020). Metabolic syndrome, non-alcoholic fatty liver disease and hepatocarcinoma. Rev. Esp. Enferm. Dig. Organo Of. Soc. Esp. Patol. Dig..

[32] Zorena K., Jachimowicz-Duda O., Ślęzak D., Robakowska M., Mrugacz M. (2020). Adipokines and obesity. Potential link to metabolic disorders and chronic complications. Int. J. Mol. Sci..

[33] Tchang B.G., Saunders K.H., Igel L.I. (2021). Best practices in the management of overweight and obesity. Med. Clin. N. Am..

[34] Wormser D., Kaptoge S., Di Angelantonio E., Wood A.M., Pennells L., Thompson A., Sarwar N., Kizer J.R., Lawlor D.A., Nordestgaard B.G. (2011). Separate and combined associations of body-mass index and abdominal adiposity with cardiovascular disease: Collaborative analysis of 58 prospective studies. Lancet.

[35] Hatem S.N. (2015). Atrial Fibrillation and Obesity: Not Just a Coincidence.

[36] Ren J., Wu N.N., Wang S., Sowers J.R., Zhang Y. (2021). Obesity cardiomyopathy: Evidence, mechanisms, and therapeutic implications. Physiol. Rev..

[37] Steele C.B., Thomas C.C., Henley S.J., Massetti G.M., Galuska D.A., Agurs-Collins T., Puckett M., Richardson L.C. (2017). Vital signs: Trends in incidence of cancers associated with overweight and obesity—United states, 2005–2014. MMWR Morb. Mortal. Wkly. Rep..

[38] Gallagher E.J., LeRoith D. (2015). Obesity and diabetes: The increased risk of cancer and cancer-related mortality. Physiol. Rev..

[39] Scappaticcio L., Maiorino M.I., Bellastella G., Giugliano D., Esposito K. (2017). Insights into the relationships between diabetes, prediabetes, and cancer. Endocrine.

[40] Aune D., Norat T., Vatten L.J. (2015). Body mass index, abdominal fatness and the risk of gallbladder disease. Eur. J. Epidemiol..

[41] Brewer C.J., Balen A.H. (2010). The adverse effects of obesity on conception and implantation. Reproduction.

[42] Bellastella G., Menafra D., Puliani G., Colao A., Savastano S. (2019). How much does obesity affect the male reproductive function?. Int. J. Obes. Suppl..

[43] Gregoriou O., Bakas P., Grigoriadis C., Creatsa M., Hassiakos D., Creatsas G. (2012). Changes in hormonal profile and seminal parameters with use of aromatase inhibitors in management of infertile men with low testosterone to estradiol ratios. Fertil. Steril..

[44] Practice Committee of the American Society for Reproductive Medicine (2021). Obesity and reproduction: A committee opinion. Fertil. Steril..

[45] Flint S.W., Čadek M., Codreanu S.C., Ivić V., Zomer C., Gomoiu A. (2016). Obesity discrimination in the recruitment process: “You’re not hired!”. Front. Psychol..

[46] Dixon J.B., Dixon M.E., O’Brien P.E. (2003). Depression in association with severe obesity: Changes with weight loss. Arch. Intern. Med..

[47] Dalamaga M., Christodoulatos G.S., Mantzoros C.S. (2018). The role of extracellular and intracellular nicotinamide phosphoribosyl-transferase in cancer: Diagnostic and therapeutic perspectives and challenges. Metabolism.

[48] Kim D.S., Scherer P.E. (2021). Obesity, diabetes, and increased cancer progression. Diabetes Metab. J..

[49] Khandekar M.J., Cohen P., Spiegelman B.M. (2011). Molecular mechanisms of cancer development in obesity. Nat. Rev. Cancer.

[50] WCRF/AICR (2018). Continuous Update Project Expert Report 2018. Body Fatness and Weight Gain and the Risk of Cancer.

[51] Jarvis D., Mitchell J.S., Law P.J., Palin K., Tuupanen S., Gylfe A., Hänninen U.A., Cajuso T., Tanskanen T., Kondelin J. (2016). Mendelian randomisation analysis strongly implicates adiposity with risk of developing colorectal cancer. Br. J. Cancer.

[52] Aune D., Navarro Rosenblatt D.A., Chan D.S.M., Vingeliene S., Abar L., Vieira A.R., Greenwood D.C., Bandera E.V., Norat T. (2015). Anthropometric factors and endometrial cancer risk: A systematic review and dose-response meta-analysis of prospective studies. Ann. Oncol. Off. J. Eur. Soc. Med. Oncol..

[53] Staiano A.E., Reeder B.A., Elliott S., Joffres M.R., Pahwa P., Kirkland S.A., Paradis G., Katzmarzyk P.T. (2012). Body mass index versus waist circumference as predictors of mortality in canadian adults. Int. J. Obes..

[54] Staunstrup L.M., Nielsen H.B., Pedersen B.K., Karsdal M., Blair J.P.M., Christensen J.F., Bager C.L. (2019). Cancer risk in relation to body fat distribution, evaluated by dxa-scans, in postmenopausal women—The prospective epidemiological risk factor (perf) study. Sci. Rep..

[55] Chen J., Du B. (2019). Novel positioning from obesity to cancer: Fto, an m6a rna demethylase, regulates tumour progression. J. Cancer Res. Clin. Oncol..

[56] Deng X., Su R., Stanford S., Chen J. (2018). Critical enzymatic functions of fto in obesity and cancer. Front. Endocrinol..

[57] Hernández-Caballero M.E., Sierra-Ramírez J.A. (2015). Single nucleotide polymorphisms of the fto gene and cancer risk: An overview. Mol. Biol. Rep..

[58] Loos R.J.F., Yeo G.S.H. (2014). The bigger picture of fto—the first gwas-identified obesity gene. Nat. Rev. Endocrinol..

[59] Mehrdad M., Doaei S., Gholamalizadeh M., Fardaei M., Fararouei M., Eftekhari M.H. (2020). Association of fto rs9939609 polymorphism with serum leptin, insulin, adiponectin, and lipid profile in overweight adults. Adipocyte.

[60] Barone I., Giordano C., Bonofiglio D., Andò S., Catalano S. (2020). The weight of obesity in breast cancer progression and metastasis: Clinical and molecular perspectives. Semin. Cancer Biol..

[61] Mauro L., Naimo G.D., Gelsomino L., Malivindi R., Bruno L., Pellegrino M., Tarallo R., Memoli D., Weisz A., Panno M.L. (2018). Uncoupling effects of estrogen receptor α on lkb1/ampk interaction upon adiponectin exposure in breast cancer. FASEB J..

[62] Booth A., Magnuson A., Fouts J., Foster M. (2015). Adipose tissue, obesity and adipokines: Role in cancer promotion. Horm. Mol. Biol. Clin. Investig..

[63] Scherer P.E. (2016). The multifaceted roles of adipose tissue—therapeutic targets for diabetes and beyond: The 2015 banting lecture. Diabetes.

[64] Di Zazzo E., Polito R., Bartollino S., Nigro E., Porcile C., Bianco A., Daniele A., Moncharmont B. (2019). Adiponectin as link factor between adipose tissue and cancer. Int. J. Mol. Sci..

[65] Brochu-Gaudreau K., Rehfeldt C., Blouin R., Bordignon V., Murphy B.D., Palin M.-F. (2010). Adiponectin action from head to toe. Endocrine.

[66] Wang G.X., Zhao X.Y., Lin J.D. (2015). The brown fat secretome: Metabolic functions beyond thermogenesis. Trends Endocrinol. Metab..

[67] Pischon T., Nimptsch K. (2016). Obesity and Risk of Cancer: An Introductory Overview.

[68] Chen X., Wang Y. (2011). Adiponectin and breast cancer. Med. Oncol..

[69] Fu S., Xu H., Liu C., Gu M., Wang Q., Zhou J., Wang Z. (2017). [role of adiponectin in prostate cancer: A preliminary study]. Zhonghua Nan Ke Xue = Natl. J. Androl..

[70] Gao Q., Zheng J., Yao X., Peng B. (2015). Adiponectin inhibits vegf-a in prostate cancer cells. Tumour Biol. J. Int. Soc. Oncodev. Biol. Med..

[71] Shrestha A., Nepal S., Kim M.J., Chang J.H., Kim S.-H., Jeong G.-S., Jeong C.-H., Park G.H., Jung S., Lim J. (2016). Critical role of ampk/foxo3a axis in globular adiponectin-induced cell cycle arrest and apoptosis in cancer cells. J. Cell. Physiol..

[72] Xing S.Q., Zhang C.G., Yuan J.F., Yang H.M., Zhao S.D., Zhang H. (2015). Adiponectin induces apoptosis in hepatocellular carcinoma through differential modulation of thioredoxin proteins. Biochem. Pharmacol..

[73] Moon H.-S., Liu X., Nagel J.M., Chamberland J.P., Diakopoulos K.N., Brinkoetter M.T., Hatziapostolou M., Wu Y., Robson S.C., Iliopoulos D. (2013). Salutary effects of adiponectin on colon cancer: In vivo and in vitro studies in mice. Gut.

[74] Otani K., Ishihara S., Yamaguchi H., Murono K., Yasuda K., Nishikawa T., Tanaka T., Kiyomatsu T., Hata K., Kawai K. (2017). Adiponectin and colorectal cancer. Surg. Today.

[75] Saxena A., Baliga M.S., Ponemone V., Kaur K., Larsen B., Fletcher E., Greene J., Fayad R. (2013). Mucus and adiponectin deficiency: Role in chronic inflammation-induced colon cancer. Int. J. Colorectal Dis..

[76] Saxena A., Chumanevich A., Fletcher E., Larsen B., Lattwein K., Kaur K., Fayad R. (2012). Adiponectin deficiency: Role in chronic inflammation induced colon cancer. Biochim. Biophys. Acta Mol. Basis Dis..

[77] Kim A.Y., Lee Y.S., Kim K.H., Lee J.H., Lee H.K., Jang S.H., Kim S.E., Lee G.Y., Lee J.W., Jung S.A. (2010). Adiponectin represses colon cancer cell proliferation via adipor1- and -r2-mediated ampk activation. Mol. Endocrinol..

[78] Guo X., Liu J., You L., Li G., Huang Y., Li Y. (2015). Association between adiponectin polymorphisms and the risk of colorectal cancer. Genet. Test. Mol. Biomark..

[79] Taliaferro-Smith L., Nagalingam A., Knight B.B., Oberlick E., Saxena N.K., Sharma D. (2013). Integral role of ptp1b in adiponectin-mediated inhibition of oncogenic actions of leptin in breast carcinogenesis. Neoplasia.

[80] Wu X., Yan Q., Zhang Z., Du G., Wan X. (2012). Acrp30 inhibits leptin-induced metastasis by downregulating the jak/stat3 pathway via ampk activation in aggressive spec-2 endometrial cancer cells. Oncol. Rep..

[81] Man K., Ng K.T.P., Xu A., Cheng Q., Lo C.M., Xiao J.W., Sun B.S., Lim Z.X.H., Cheung J.S., Wu E.X. (2010). Suppression of liver tumor growth and metastasis by adiponectin in nude mice through inhibition of tumor angiogenesis and downregulation of rho kinase/ifn-inducible protein 10/matrix metalloproteinase 9 signaling. Clin. Cancer Res..

[82] Cui E., Guo H., Shen M., Yu H., Gu D., Mao W., Wang X. (2018). Adiponectin inhibits migration and invasion by reversing epithelial-mesenchymal transition in non-small cell lung carcinoma. Oncol. Rep..

[83] Kahn C.R., Wang G., Lee K.Y. (2019). Altered adipose tissue and adipocyte function in the pathogenesis of metabolic syndrome. J. Clin. Investig..

[84] Orgel E., Mittelman S.D. (2013). The links between insulin resistance, diabetes, and cancer. Curr. Diabetes Rep..

[85] Wang M., Zhao J., Zhang L., Wei F., Lian Y., Wu Y., Gong Z., Zhang S., Zhou J., Cao K. (2017). Role of tumor microenvironment in tumorigenesis. J. Cancer.

[86] Ouchi N., Parker J.L., Lugus J.J., Walsh K. (2011). Adipokines in inflammation and metabolic disease. Nat. Rev. Immunol..

[87] Park J., Euhus D.M., Scherer P.E. (2011). Paracrine and endocrine effects of adipose tissue on cancer development and progression. Endocr. Rev..

[88] Kumar S., Behl T., Sachdeva M., Sehgal A., Kumari S., Kumar A., Kaur G., Yadav H.N., Bungau S. (2021). Implicating the effect of ketogenic diet as a preventive measure to obesity and diabetes mellitus. Life Sci..

[89] Kazamel M., Stino A.M., Smith A.G. (2021). Metabolic syndrome and peripheral neuropathy. Muscle Nerve.

[90] Hicks C.W., Selvin E. (2019). Epidemiology of peripheral neuropathy and lower extremity disease in diabetes. Curr. Diabetes Rep..

[91] Feldman E.L., Nave K.A., Jensen T.S., Bennett D.L.H. (2017). New horizons in diabetic neuropathy: Mechanisms, bioenergetics, and pain. Neuron.

[92] Gonçalves N.P., Vægter C.B., Pallesen L.T. (2018). Peripheral glial cells in the development of diabetic neuropathy. Front. Neurol..

[93] Feldman E.L., Callaghan B.C., Pop-Busui R., Zochodne D.W., Wright D.E., Bennett D.L., Bril V., Russell J.W., Viswanathan V. (2019). Diabetic neuropathy. Nat. Rev. Dis. Primers.

[94] Bodman M.A., Varacallo M. (2021). Peripheral diabetic neuropathy. Statpearls.

[95] Jin H.Y., Park T.S. (2018). Role of inflammatory biomarkers in diabetic peripheral neuropathy. J. Diabetes Investig..

[96] Zafeiri M., Tsioutis C., Kleinaki Z., Manolopoulos P., Ioannidis I., Dimitriadis G. (2021). Clinical characteristics of patients with co-existent diabetic peripheral neuropathy and depression: A systematic review. Exp. Clin. Endocrinol. Diabetes Off. J. Ger. Soc. Endocrinol. Ger. Diabetes Assoc..

[97] Sloan G., Shillo P., Selvarajah D., Wu J., Wilkinson I.D., Tracey I., Anand P., Tesfaye S. (2018). A new look at painful diabetic neuropathy. Diabetes Res. Clin. Pract..

[98] Cole J.B., Florez J.C. (2020). Genetics of diabetes mellitus and diabetes complications. Nat. Rev. Nephrol..

[99] Braffett B.H., Gubitosi-Klug R.A., Albers J.W., Feldman E.L., Martin C.L., White N.H., Orchard T.J., Lopes-Virella M., Lachin J.M., Pop-Busui R. (2020). Risk factors for diabetic peripheral neuropathy and cardiovascular autonomic neuropathy in the diabetes control and complications trial/epidemiology of diabetes interventions and complications (dcct/edic) study. Diabetes.

[100] Liu X., Xu Y., An M., Zeng Q. (2019). The risk factors for diabetic peripheral neuropathy: A meta-analysis. PLoS ONE.

[101] Seyedizadeh S.H., Cheragh-Birjandi S., Hamedi Nia M.R. (2020). The effects of combined exercise training (resistance-aerobic) on serum kinesin and physical function in type 2 diabetes patients with diabetic peripheral neuropathy (randomized controlled trials). J. Diabetes Res..

[102] Zilliox L.A., Russell J.W. (2019). Physical activity and dietary interventions in diabetic neuropathy: A systematic review. Clin. Auton. Res. Off. J. Clin. Auton. Res. Soc..

[103] Rumora A.E., Savelieff M.G., Sakowski S.A., Feldman E.L. (2019). Disorders of mitochondrial dynamics in peripheral neuropathy: Clues from hereditary neuropathy and diabetes. Int. Rev. Neurobiol..

[104] Spallone V., Ciccacci C., Latini A., Borgiani P. (2021). What is in the field for genetics and epigenetics of diabetic neuropathy: The role of micrornas. J. Diabetes Res..

[105] Cheng Y., Liu J., Luan Y., Liu Z., Lai H., Zhong W., Yang Y., Yu H., Feng N., Wang H. (2019). Sarm1 gene deficiency attenuates diabetic peripheral neuropathy in mice. Diabetes.

[106] Shillo P., Selvarajah D., Greig M., Gandhi R., Rao G., Wilkinson I.D., Anand P., Tesfaye S. (2019). Reduced vitamin d levels in painful diabetic peripheral neuropathy. Diabet. Med. A J. Br. Diabet. Assoc..

[107] Xue T., Zhang X., Xing Y., Liu S., Zhang L., Wang X., Yu M. (2021). Advances about immunoinflammatory pathogenesis and treatment in diabetic peripheral neuropathy. Front. Pharmacol..

[108] Freedland S.J., Aronson W.J. (2009). Words of wisdom. Re: Weight loss with a low-carbohydrate, mediterranean, or low-fat diet. N. Engl. J. Med. 2008, 359, 229–241. Eur. Urol..

[109] Larsen R.N., Mann N.J., Maclean E., Shaw J.E. (2011). The effect of high-protein, low-carbohydrate diets in the treatment of type 2 diabetes: A 12 month randomised controlled trial. Diabetologia.

[110] Yumuk V., Tsigos C., Fried M., Schindler K., Busetto L., Micic D., Toplak H., Obesity Management Task Force of the European Association for the Study of Obesity (2015). European guidelines for obesity management in adults. Obes. Facts.

[111] Wadden T.A., West D.S., Neiberg R.H., Wing R.R., Ryan D.H., Johnson K.C., Foreyt J.P., Hill J.O., Trence D.L., Vitolins M.Z. (2009). One-year weight losses in the look ahead study: Factors associated with success. Obesity.

[112] Leitner D.R., Fruhbeck G., Yumuk V., Schindler K., Micic D., Woodward E., Toplak H. (2017). Obesity and type 2 diabetes: Two diseases with a need for combined treatment strategies - easo can lead the way. Obes. Facts.

[113] Geliebter A., Ochner C.N., Dambkowski C.L., Hashim S.A. (2014). Obesity-related hormones and metabolic risk factors: A randomized trial of diet plus either strength or aerobic training versus diet alone in overweight participants. J. Diabetes Obes..

[114] Willis L.H., Slentz C.A., Bateman L.A., Shields A.T., Piner L.W., Bales C.W., Houmard J.A., Kraus W.E. (2012). Effects of aerobic and/or resistance training on body mass and fat mass in overweight or obese adults. J. Appl. Physiol..

[115] Thibault R., Huber O., Azagury D.E., Pichard C. (2016). Twelve key nutritional issues in bariatric surgery. Clin. Nutr..

[116] Jackson V.M., Breen D.M., Fortin J.P., Liou A., Kuzmiski J.B., Loomis A.K., Rives M.L., Shah B., Carpino P.A. (2015). Latest approaches for the treatment of obesity. Expert Opin. Drug Discov..

[117] Wang Z., Xiong H., Ren T.Y.S. (2021). Repair of damaged pancreatic beta cells: New hope for a type 2 diabetes reversal?. J Transl Int Med.

[118] Tak Y.J., Lee S.Y. (2021). Long-term efficacy and safety of anti-obesity treatment: Where do we stand?. Curr. Obes. Rep..

[119] Khera R., Murad M.H., Chandar A.K., Dulai P.S., Wang Z., Prokop L.J., Loomba R., Camilleri M., Singh S. (2016). Association of pharmacological treatments for obesity with weight loss and adverse events: A systematic review and meta-analysis. JAMA.

[120] Davies M.J., Bergenstal R., Bode B., Kushner R.F., Lewin A., Skjoth T.V., Andreasen A.H., Jensen C.B., DeFronzo R.A., Group N.N.S. (2015). Efficacy of liraglutide for weight loss among patients with type 2 diabetes: The scale diabetes randomized clinical trial. JAMA.

[121] Yanovski S.Z., Yanovski J.A. (2014). Long-term drug treatment for obesity: A systematic and clinical review. JAMA.

[122] Daubresse M., Alexander G.C. (2015). The uphill battle facing antiobesity drugs. Int. J. Obes..

[123] Fried M., Yumuk V., Oppert J.M., Scopinaro N., Torres A.J., Weiner R., Yashkov Y., Fruhbeck G., European Association for the Study of Obesity, International Federation for the Surgery of Obesity-European Chapter (2013). Interdisciplinary european guidelines on metabolic and bariatric surgery. Obes. Facts.

[124] Jabbour G., Salman A. (2021). Bariatric surgery in adults with obesity: The impact on performance, metabolism, and health indices. Obes. Surg..

[125] Esteghamati A., Mazaheri T., Vahidi Rad M., Noshad S. (2015). Complementary and alternative medicine for the treatment of obesity: A critical review. Int. J. Endocrinol. Metab..

[126] Liu Y., Sun M., Yao H., Liu Y., Gao R. (2017). Herbal medicine for the treatment of obesity: An overview of scientific evidence from 2007 to 2017. Evid. Based Complement. Alternat. Med..

[127] Zhou Q., Chang B., Chen X.Y., Zhou S.P., Zhen Z., Zhang L.L., Sun X., Zhou Y., Xie W.Q., Liu H.F. (2014). Chinese herbal medicine for obesity: A randomized, double-blinded, multicenter, prospective trial. Am. J. Chin. Med..

[128] Gao Y., Wang Y., Zhou J., Hu Z., Shi Y. (2020). Effectiveness of electroacupuncture for simple obesity: A systematic review and meta-analysis of randomized controlled trials. Evid. Based Complement. Alternat. Med..

[129] Darbandi M., Darbandi S., Owji A.A., Mokarram P., Mobarhan M.G., Fardaei M., Zhao B., Abdi H., Nematy M., Safarian M. (2014). Auricular or body acupuncture: Which one is more effective in reducing abdominal fat mass in iranian men with obesity: A randomized clinical trial. J. Diabetes Metab. Disord..

[130] Wang D., Rong P.J., Li S.Y., Wang Y.F., Zhang Z.X., He J.K. (2020). [bibliometric analysis on clinical trials of auricular acupuncture treatment of obesity]. Zhen Ci Yan Jiu.

